# Resting state activity and connectivity of the nucleus basalis of Meynert and globus pallidus in Lewy body dementia and Parkinson's disease dementia

**DOI:** 10.1016/j.neuroimage.2020.117184

**Published:** 2020-11-01

**Authors:** James Gratwicke, Ashwini Oswal, Harith Akram, Marjan Jahanshahi, Marwan Hariz, Ludvic Zrinzo, Tom Foltynie, Vladimir Litvak

**Affiliations:** a Department of Clinical and Movement Neurosciences, UCL Institute of Neurology and The National Hospital for Neurology and Neurosurgery, Queen Square, London, UK; b Nuffield Department of Clinical Neurosciences, John Radcliffe Hospital, Oxford, UK; c Wellcome Centre for Human Neuroimaging, UCL Institute of Neurology, 12 Queen Square, London, UK

**Keywords:** Human, Basal ganglia, Network, Pallidum, Basal forebrain

## Abstract

Parkinson's disease dementia (PDD) and dementia with Lewy bodies (DLB) are two related diseases which can be difficult to distinguish. There is no objective biomarker which can reliably differentiate between them. The synergistic combination of electrophysiological and neuroimaging approaches is a powerful method for interrogation of functional brain networks in vivo. We recorded bilateral local field potentials (LFPs) from the nucleus basalis of Meynert (NBM) and the internal globus pallidus (GPi) with simultaneous cortical magnetoencephalography (MEG) in six PDD and five DLB patients undergoing surgery for deep brain stimulation (DBS) to look for differences in underlying resting-state network pathophysiology. In both patient groups we observed spectral peaks in the theta (2–8 Hz) band in both the NBM and the GPi. Furthermore, both the NBM and the GPi exhibited similar spatial and spectral patterns of coupling with the cortex in the two disease states. Specifically, we report two distinct coherent networks between the NBM/GPi and cortical regions: (1) a theta band (2–8 Hz) network linking the NBM/GPi to temporal cortical regions, and (2) a beta band (13–22 Hz) network coupling the NBM/GPi to sensorimotor areas. We also found differences between the two disease groups: oscillatory power in the low beta (13–22Hz) band was significantly higher in the globus pallidus in PDD patients compared to DLB, and coherence in the high beta (22–35Hz) band between the globus pallidus and lateral sensorimotor cortex was significantly higher in DLB patients compared to PDD. Overall, our findings reveal coherent networks of the NBM/GPi region that are common to both DLB and PDD. Although the neurophysiological differences between the two conditions in this study are confounded by systematic differences in DBS lead trajectories and motor symptom severity, they lend support to the hypothesis that DLB and PDD, though closely related, are distinguishable from a neurophysiological perspective.

## 1 Introduction

Dementia with Lewy bodies (DLB) and Parkinson's disease dementia (PDD) are two of the most common neurodegenerative dementias (Aarsland and Kurz, 2010; Jellinger, 2018). They share a common clinical phenotype, characterized by prominent impairments in executive, attentional and perceptive functions, with associated motor parkinsonism and psychotic symptoms (Emre et al., 2007; Gratwicke et al., 2015; McKeith et al., 2017). The presence of cortical Lewy bodies composed of alpha-synuclein is also the common pathological hallmark of both conditions (Horvath et al., 2013; Hurtig et al., 2000), and both are associated with prominent central nervous system cholinergic dysfunction (Shimada et al., 2009). Despite these similarities, significant differences exist in the timing and severity of motor symptoms, psychotic symptoms and co-existent Alzheimer's type pathology, and therefore whether DLB and PDD represent two manifestations of the same condition remains controversial (Goldman et al., 2014). No objective biomarker has yet been found which can reliably differentiate the two conditions (Bibl et al., 2006; Mollenhauer et al., 2008; Stefani et al., 2012).

We recently investigated the tolerability and potential symptomatic effects of low frequency deep brain stimulation (DBS) of the nucleus basalis of Meynert (NBM) in patients with both DLB (Gratwicke et al., 2020) and PDD (Gratwicke et al., 2018). The NBM provides the main source of cholinergic innervation to the entire cortical mantle (‘corticopetal’ innervation) and is strongly implicated in arousal, attention, memory and perceptive functions (Gratwicke et al., 2013; Mesulam and Geula, 1988; Mufson et al., 2003). Degeneration of this nucleus has been shown to be key in the pathogenesis of both DLB and PDD (Choi et al., 2012; Grothe et al., 2014; Ray et al., 2018; Whitehouse et al., 1983) and neuromodulation of NBM and its residual connections has, therefore, been proposed as a potential treatment (Gratwicke et al., 2013; Hardenacke et al., 2012; Kuhn et al., 2015). To allow our patients to potentially benefit from both cognitive and motor effects of DBS, the electrodes in both trials were sited so that their most ventral contacts lay in NBM and their most dorsal pair of contacts lay in the globus pallidus internus (GPi) respectively. GPi DBS is a well recognised treatment for parkinsonian motor symptoms such as bradykinesia and rigidity (Foltynie and Hariz, 2010; Odekerken et al., 2012).

Our studies afforded us the unique opportunity to record bilateral local field potentials (LFPs) from both the NBM and the GPi region, while simultaneously recording cortical magnetoencephalography (MEG). Through this synergistic combination of electrophysiological and imaging techniques we were able to directly characterize resting state activity and cortical connectivity of these regions. As the PDD and DLB patients in the studies were of matched dementia severity this, therefore, allowed us to examine differences in cognitive and motor network activity and connectivity between these two disease entities in vivo, and thereby investigate the relationship between them from a functional network perspective.

## 2 Methods

### 2.1 Patients and surgery

A total of eleven patients (six with PDD and five with DLB) participated in the clinical trials. Of these all six PDD patients and four DLB patients participated in simultaneous recordings of intracranial LFPs and MEG. One patient with DLB was unable (due to fatigue) to participate in simultaneous LFP-MEG recordings, but did participate in a different session of simultaneous LFP and EEG recordings (see below under heading “Analysis of LFP power”).

Our surgical procedure for NBM DBS implantation was almost identical in both trials and has been previously reported (Gratwicke et al., 2018). The only difference between the trials in this respect was that in the DLB trial we selected a target for our deepest electrode contacts which was a few millimeters more anteromedial in the Ch4i subsector of NBM (see Gratwicke et al., 2013 for detailed description of the subarchitecture of the human NBM) compared to that used in the PDD trial. This was done to optimise location of the deepest contacts within NBM for the purposes of the DLB trial (where concurrent optimal targeting of the posteroventral GPi was not so clinically important due to the lower motor symptom burden in these patients). Thus, in some patients (more commonly in the DLB group), the top contacts were not inside GPi but in the dorsal external globus pallidus (GPe).

We, therefore, refer to *GPi region* in the descriptions below. Combined LFP and MEG/EEG recordings were made in all patients during the period of electrode externalisation on the ward, between electrode implantation and stimulator implantation (a period of 4-7 days). Clinical characteristics of the individual patients are presented in Table 1, and the reconstructed co-ordinates of the individual DBS electrode contacts in each hemisphere normalised to the right hemisphere template space (see below) are given in Table 2.

**Table 1:** Baseline clinical characteristics of the study sample.

Patient	Diagnosis	Sex	Age at surgery (yrs)	Disease duration (yrs)	UPDRS Part III motor score OFF medication (range 0–132)	Hoehn & Yahr stage	MMSE	Mattis Dementia Rating Scale 2 (Raw, Scaled)	Daily levodopa equivalent dose (LED, mg/day)	Dailytotal cholinesterase inhibitor dose (mg/day)
A	PDD	M	61	14	56	3	25	124, Scaled 5 (moderately impaired)	500	3
B	PDD	M	65	11	50	2	24	116, Scaled 3 (severely impaired)	923.25	4.6
C	PDD	M	75	11	39	2	25	126, Scaled 6 (mildly impaired)	670	6
D	PDD	M	73	15	62	3	25	110, Scaled 3 (severely impaired)	380	6
E	PDD	M	46	10	49	2	22	108, Scaled 2 (severely impaired)	575	12
F	PDD	M	71	15	24	3	21	101, Scaled 2 (severely impaired)	833	9
PDD Group Mean (SD)			65.17 (10.74)	12.67 (2.25)	46.67 (13.50)	2.50 (0.55)	23.67 (1.75)	114.17 (9.68)	646.88 (204.71)	6.77 (3.24)
G	DLB	M	65	5	35	3	23	126, Scaled 5 (moderately impaired)	187.5	10
H	DLB	M	73	4	42	2	23	121, Scaled 4 (moderately impaired)	375	9
I	DLB	F	73	10	24	3	21	123, Scaled 5 (moderately impaired)	1000	9.5
J	DLB	M	75	3	20	2	24	82, Scaled 2 (severely impaired)	62.5	10
K	DLB	M	73	4	16	2	24	125, Scaled 5 (moderately impaired)	0	10
DLB Group Mean (SD)			71.80 (3.90)	5.20 (2.77)	27.40 (10.81)	2.40 (0.55)	23.00 (1.22)	115.40 (18.77)	325.00 (403.60)	9.70 (0.45)

Dementia duration was estimated by examining the patient's medical notes and collateral history from the caregiver to determine the time at which cognitive decline began to interfere with normal occupational or social function. The Mattis Dementia Rating Scale 2 Scaled score is corrected for age but not education. SD = standard deviation. UPDRS = Movement Disorders Society Unified Parkinson's Disease Rating Scale. LED calculation as per protocol in Tomlinson et al., 2010.

**Table 2:** Stereotactic coordinates of individual DBS contacts transformed to common template space

Patient	Contact	Right hemisphere coordinates	Left hemisphere coordinates (transformed to the right)
	3	19.98, −1.25, −1.35	19.35, −2.51, −4.13
A	2	19.61, −2.09, −4.95	19.06, −2.69, −7.56
	1	19.22, −2.86, −8.63	18.78, −2.88, −11.15
	0	18.83, −3.60,−12.50	18.38, −3.11, −14.89
	3	20.18, −6.83,0.12	21.29, −6.02, −0.28
B	2	20.20, −7.22, −2.23	21.45, −6.47, −2.61
	1	20.23, −7.58, −4.61	21.61, −6.85, −4.98
	0	20.26, −7.91,−7.02	21.77, −7.18, −7.36
	3	18.88, −5.12, −3.04	20.64, −5.44, −3.04
C	2	18.61, −5.69, −5.24	20.44, −5.98, −5.25
	1	18.34, −6.27, −7.47	20.22, −6.52, −7.47
	0	18.07, −6.84, −9.72	20.01, −7.05, −9.66
	3	22.09, −5.12, −4.63	22.35, −3.30, −4.24
D	2	22.15, −5.71, −6.83	22.17, −3.95, −6.44
	1	22.26, −6.27, −9.16	22.02, −4.58, −8.72
	0	22.41, −6.81, −11.60	21.87, −5.21, −11.07
	3	21.73, −6.43, −3.96	23.13, −5.34, −3.26
E	2	21.83, −7.05, −6.25	23.12, −5.81, −5.49
	1	21.91, −7.65, −8.54	23.10, −6.27, −7.70
	0	22.00, −8.21, −10.85	23.09, −6.73, −9.88
	3	21.23, −5.39, −2.92	21.83, −6.90, −2.30
F	2	21.20, −5.80, −5.23	21.8, −7.11, −4.71
	1	21.17, −6.21, −7.55	21.80, −7.27, −7.13
	0	21.13, −6.61, −9.88	21.82, −7.41, −9.53
	3	19.80, −0.91, −3.61	23.51, −3.14, −4.09
G	2	19.87, −2.43, −5.49	23.57, −4.59, −5.98
	1	19.95, −3.94, −7.39	23.61, −6.02, −7.86
	0	20.03, −5.41, −9.30	23.64, −7.43, −9.72
	3	20.82, 0.14, −6.21	23.55, 1.49, −9.18
H	2	20.49, −1.43, −7.90	23.30, −0.22, −10.87
	1	20.17, −2.97, −9.57	23.02, −1.92, −12.55
	0	19.84, −4.48, −11.24	22.68, −3.54, −14.20
	3	19.56, −2.22, −3.01	20.81, −2.87, −4.51
_I_	2	19.09, −2.69, −5.43	20.44, −3.09, −7.06
	1	18.59, −3.11, −7.87	20.04, −3.30, −9.61
	0	18.05, −3.49, −10.32	19.57, −3.52, −12.19
	3	19.27, −1.99, −3.64	20.08, −2.25, −2.76
J	2	19.04, −2.28, −6.28	19.69, −2.64, −5.35
	1	18.79, −2.54, −8.95	19.24, −2.98, −7.97
	0	18.52, −2.77, −11.64	18.70, −3.31, −10.59
	3	19.94, −3.80, −5.57	20.53, −2.84, −4.98
K	2	19.51, −4.27, −8.04	20.26, −3.68, −7.32
	1	19.07, −4.71, −10.53	19.92, −4.54, −9.60
	0	18.63, −5.13, −13.04	19.53, −5.43, −11.85

**Fig. 1:**
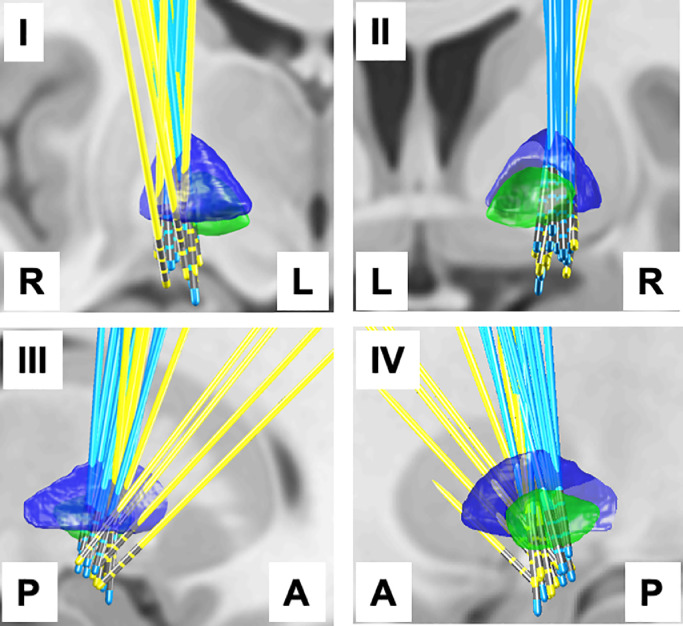
DBS electrode locations reconstructed and transformed to the template space using Lead-DBS. The left electrodes were transformed to the right hemisphere for the purposes of group analysis and visualisation. The same scene is shown from four directions: axial view from the front (I), axial view from the back (II), sagittal view from right (III), sagittal view from the left (IV). To aid interpretation, left-L, right-R, anterior-A and posterior-P directions are indicated in the images. The electrodes of the PDD group are cyan and the electrodes of the DLB group are yellow. GPe boundaries are rendered in blue and GPi boundaries in green based on the DISTAL atlas (Ewert et al., 2018).

Stereotactic coordinates of the individual DBS contacts in each patient reconstructed with Lead-DBS (see Methods) are presented in format (x,y,z) in reference to the midcommissural point of the anterior commissure - posterior commissure plane. In each patient contact 3 is the most dorsal (corresponding to the GPi region) and contact 0 is the most ventral (corresponding to NBM). These coordinates correspond exactly to Fig. 1.

### 2.2 Study approval

The study was sponsored by UCL and performed at the National Hospital for Neurology and Neurosurgery, London, UK. Ethical approval and consent for the combined LFP and MEG/EEG recordings were included in the main ethics applications and consent forms respectively for the two clinical trials. Both trials conformed to the Seoul revision of the Declaration of Helsinki (2008) and Good Clinical Practice guidelines and were approved by the East of England Research Ethics Committee. Prior to providing written informed consent, all enrolled participants were evaluated by an independent neuropsychologist to ensure they have capacity do so.

### 2.3 Experimental paradigm

Patients took part in two experimental sessions. One session consisted of combined LFP and EEG recordings. The second session performed on a different day included simultaneous LFP and MEG recordings. All recordings were performed in the resting state.

Both recording sessions were performed during the daytime with the patients having taken their usual doses of both levodopa and acetylcholinesterase (AChEI) medications beforehand. Details of the relative doses of these medications amongst individual patients are provided in Table 1. While sitting comfortably patients were instructed to remain still with their eyes open for 3 minutes. They were instructed to focus their gaze on a fixation point. A neurologist was present during all experiments (inside the magnetically shielded room in the case of the MEG recordings) to monitor the patients’ wellbeing and to ensure that they remained awake throughout the recordings.

### 2.4 Data acquisition

Combined LFP-EEG recordings were done using a Porti 32-channel amplifier (TMSi, Oldenzaal, The Netherlands). The data were recorded with the average reference, sampled at 2048 Hz and converted offline to a bipolar montage. Only LFP data were used in the present analysis.

MEG recordings were performed with a 275 channel CTF system (VSM MedTech Ltd., Vancouver, Canada). MEG data were sampled at 2400 Hz and stored to disk for subsequent offline analyses. Head location in the MEG scanner was monitored using three head position indicator (HPI) coils attached to the subject's nasion and both pre-auricular points. For each patient, head location was recorded continuously throughout the experiment. Loss of head tracking occurred intermittently in some patients, likely due to metal artefacts from their implanted DBS hardware disrupting the head tracking function of the sensors. The head tracking information was corrected by interpolation based on the valid segments as we have previously described (Oswal et al., 2016b).

Simultaneously with MEG, bilateral NBM and GPi LFPs, electro-oculographic (EOG) and electromyographic (EMG) signals were recorded using a battery-powered and optically isolated BrainAmp system (Brain Products GmbH, Gilching, Germany). As this is a separate recording system to the main MEG system, fusion of the LFP and MEG data with minimal timing distortions is a challenge. To facilitate this, a common synchronisation signal was recorded on both systems – the signal used was random white noise because it can only be matched in a unique way. We have previously published a detailed description of this methodology (Oswal et al., 2016b).

Six intracranial LFP channels, each corresponding to the potential difference between adjacent pairs of DBS contacts (R01, R12, R23, L01, L12, L23) were recorded using a bipolar amplifier (BrainAmp ExG). Using the bipolar amplifier in this experiment was motivated by further experiments conducted with these patients in the same sitting using active DBS stimulation during MEG (not reported here) in which such an amplifier was necessary. EMG data were recorded from tendons of the right and left first dorsal interosseous muscles to serve as references for movement artefact in MEG. Recorded electrophysiological signals were amplified (X 50,000), hardware filtered (1.0–600 Hz), sampled at 2400 Hz (MEG) and 2500 Hz (LFP-EMG) and stored to disk.

### 2.5 Data pre-processing

The data were analysed in MATLAB (The Mathworks, Inc, Natick, MA) using custom scripts in conjunction with the SPM12 (http://www.fil.ion.ucl.ac.uk/spm/, (Litvak et al., 2011b)) and FieldTrip (http://www.fieldtriptoolbox.org/, (Oostenveld et al., 2011)) toolboxes.

We combined data from the LFP-EEG and LFP-MEG sessions for LFP power analysis to be able to incorporate all the patients including the one who did not undergo MEG recordings. In one additional PDD patient LFP-EEG recordings were of poor quality and for this patient, only data from LFP-MEG session were used. For this analysis, the data were converted to SPM format, converted to bipolar montage (for LFP-EEG data only), downsampled to 300Hz and filtered with a 1Hz high-pass filter and notch filters at the line noise frequency (50Hz) and its harmonics. All filters were 5th order, zero-phase Butterworth filters. The data were then epoched into 1 sec trials and trials containing artefacts were detected by thresholding and removed from analysis. The rejection threshold was individually adjusted for each patient based on visual inspection of the data.

The pre-processing of MEG-LFP data for coherence analysis was similar except the LFP data had been recorded in a bipolar fashion at hardware level and therefore there was no need for offline conversion to a bipolar montage. The LFP was resampled to 2400Hz to match the sampling rate of MEG and the two datasets were then fused using the white noise recorded on both systems for alignment (Oswal et al., 2016b). The data were filtered with 1Hz high-pass and notch filters and epoched into 3.41 sec trials consistent with our previous studies of rest coherence (Litvak et al., 2011a; Neumann et al., 2015). Trials with artefacts in the LFP recording were rejected by thresholding as described above.

### 2.6 Analysis of LFP power

Power spectra were estimated from LFP data with 1 sec epoch length for 1–100 Hz at 1 Hz resolution using the Fast Fourier Transform with the Hanning taper and averaged across epochs. To make the spectra more comparable across subjects and isolate their oscillatory component we used the Fitting Oscillations and One-Over F algorithm (FOOOF, https://github.com/fooof-tools/fooof, (Haller et al., 2018)). This algorithm parameterises the log-spectrum as a sum of background 1/f noise and oscillatory peaks modelled as sums of Gaussians. We only used it to fit and subtract the background component. The fit was done in the 5-95 Hz range after interpolating over 47–53Hz range affected by line noise. Good quality of the fit at least up to 45Hz was visually verified for each spectrum. We then subtracted the estimated background component from the original log-spectrum and averaged the corrected log-power spectra across recording sessions weighted by the number of epochs in the respective session. We then compared the bottom channel corresponding primarily to the NBM and the top channel corresponding primarily to the GPi region.

### 2.7 Analysis of LFP-MEG coherence

One key aim of this experiment was to study possible functional connectivity between the GPi and NBM and distant cortical regions, and to compare these relationships between the two disease entities. Functional connectivity can be assessed through the statistical relationship of activity signals occurring in two distant brain regions over a discrete time interval. Coherence is one way of measuring this. It provides a frequency-domain measure of the linear phase and amplitude relationships between two signals, bounded between 0 and 1 (Buzsáki and Draguhn, 2004; Thatcher et al., 1986). In other words, coherence is the frequency domain counterpart of a cross-correlation in the time domain. Coherent oscillatory activity between distant neural populations is believed to play an important role in their communication, and implies a functional relationship between the two areas, although it does not provide any information about the directionality of coupling (Bastos et al., 2015; Fries, 2005).

Coherence was first calculated at the sensor level, between each LFP channel and each MEG channel in order to define frequency bands of significant coherence within each patient (Litvak et al., 2011a). Coherence was computed in the 1–45Hz range, with 2.5Hz resolution. Scalp maps of coherence for each frequency bin were linearly interpolated to produce a 2D image. The resulting images were stacked to produce a 3D image with two spatial and one frequency dimension (Kilner and Friston, 2010). To determine significant regions within these images, they were compared with null (surrogate) data in which any coherence was destroyed: ten surrogate coherence images were generated from the same MEG data but with the order of LFP trials shuffled. The original and surrogate images were smoothed with a Gaussian kernel (10 mm × 10 mm × 2.5 Hz) to ensure conformance to the assumptions of random field theory. They were then subjected to a one-tailed independent samples *t*-test with equal variance assumption in SPM12 (thresholded at *p*<0.01 family-wise error corrected at the peak level) to identify significant regions in sensor space and frequency. For each individual LFP channel, this provided frequency ranges where there was significant sensor-level coherence with MEG. These results were summarised as a histogram where for each frequency bin, the number of times this bin was included in a significant coherence cluster was shown.

With this information in hand, coherence was then analysed at the source level, between each LFP channel and a 3D grid of points representing spatial locations within the brain, in order to locate coherent cortical sources. This employed the dynamic imaging of coherent sources (DICS) beamforming method (Gross et al., 2001) implemented in the DAiSS toolbox for SPM12 (https://github.com/spm/DAiSS). Beamforming has previously been shown to be effective at suppressing artefacts generated by the ferromagnetic cables connecting the DBS electrodes to the recording equipment (Litvak et al., 2010).

The forward computation was done using the single shell model (Nolte, 2003) based on an inner skull mesh derived from inverse-normalising a canonical mesh to each individual patient's pre-operative structural MRI image (Mattout et al., 2007). The source space was a 5 mm grid bounded by the inner skull surface. To be able to compare coherence topographies between frequency bands each image was normalised by that image's average value across vertices. Values at the grid points were then linearly interpolated to produce volumetric images with 2 mm resolution and saved in the Neuroimaging Informatics Technology Initiative (NIfTI) format for statistical analysis in SPM12.

To identify the cortical areas consistently coherent with the NBM and the GPi region respectively, the DICS images were computed for four pre-defined bands whose exact boundaries were informed by the sensor-level analysis: theta (2–8Hz), alpha (8–13Hz), low beta (13–22Hz) and high beta (22–35Hz) and subjected to statistical analysis in SPM using a general linear model (GLM) based approach. All images corresponding to left LFP channels were flipped across the mid-sagittal plane to allow comparison of ipsilateral and contralateral sources regardless of original side. The images were subjected to a one-way ANOVA to test for the effect of the frequency band. Regressors for subject and side were included as confounds.

We then performed additional two-way ANOVAs within each band for which significant band-specific source coherence was identified. In these ANOVAs only images from the top and bottom contact pairs were included separately for DLB and PDD patients and we tested for the effects of disease and contact and their interaction. The analysis for each band was restricted to the region of interest defined by the mask of the significant coherence in the corresponding band from the between-band ANOVA.

All the reported findings are significant with family-wise error correction at the peak level (*p*<0.05).

### 2.8 Reconstruction of electrode locations

We used the Lead-DBS toolbox (http://www.lead-dbs.org/, (Horn and Kühn, 2015)) to reconstruct the contact locations. Post-operative T2 and T1 images were co-registered to pre-operative T1 scan using linear registration in SPM12 (Friston et al., 2007). Pre- (and post-) operative acquisitions were spatially normalized into MNI_ICBM_2009b_NLIN_ASYM space based on preoperative T1 using the Unified Segmentation Approach as implemented in SPM12 (Ashburner and Friston, 2005). DBS electrode localizations were corrected for brain shift in postoperative acquisitions by applying a refined affine transform calculated between pre- and post-operative acquisitions that were restricted to a subcortical area of interest as implemented in the brain shift correction module of Lead-DBS software. The electrodes were then manually localized based on post-operative acquisitions using a tool in Lead-DBS specifically designed for this task. The resulting locations were verified by an expert neurosurgeon.

### 2.9 Data and code availability

The code used for data analyses specific to present paper is available at https://github.com/vlitvak/nbm_gpi_paper/ . The raw data will be shared subject to limitations of the ethical approval and the Wellcome Centre for Human Neuroimaging data sharing policy https://www.fil.ion.ucl.ac.uk/about/open-science/data-sharing/.

## 3 Results

### 3.1 Patient characteristics

Between October 2012 and February 2016, we completed recordings in the six PDD and five DLB patients enrolled in our two clinical trials. Further details regarding diagnosis and recruitment may be found in the corresponding clinical manuscripts (Gratwicke et al., 2020, 2018). Clinical characteristics and the stereotactic co-ordinates of the most ventral and dorsal DBS electrode contacts in each hemisphere are detailed in Tables 1 and 2 respectively. All patients were male apart from one DLB patient. The DLB patients were slightly older than the PDD patients (group mean [SD] ages 71.80 [3.90] years vs. 65.17 [10.74] years respectively). As expected, disease duration was longer in the PDD patient group compared to the DLB patient group (12.67 [2.25] years vs. 5.20 [2.77] years respectively) reflecting the delayed onset of dementia in relation to motor symptoms in the former. Both groups were well matched for dementia severity on both the Mini Mental State Examination (PDD patients 23.67 [1.75] points, DLB patients 23.00 [1.22] points) and the more detailed Mattis Dementia Rating Scale 2 (PDD patients 114.17 [9.68] points, DLB patients 115.40 [18.77] points). Nevertheless, DLB patients were being treated with larger doses of AChEI medication for their cognitive symptoms (9.70 [0.45] mg/day vs. 6.77 [3.24] mg/day in the PDD patients). As expected, motor symptom severity was greater in the PDD patients than the DLB patients (Unified Parkinson's Disease Rating Scale Part III motor score off medication 46.67 [13.50] points vs 27.40 [10.81] points respectively), and this was reflected in the fact that the PDD patients were being treated with higher doses of levodopa therapy compared to the DLB patients (646.88 [204.71] mg/day vs. 325.00 [403.60] mg/day respectively). Motor symptoms were present bilaterally in both patient groups (Hoehn and Yahr stage, PDD patients 2.5 [0.55] points, DLB patients 2.40 [0.55] points).

### 3.2 Electrode locations

Fig. 1 shows the electrode locations for PDD (blue) and DLB (yellow) patients. The left hemisphere electrodes have been transformed to the right using non-linear transform implemented in Lead-DBS, therefore, the combined electrode locations from both hemispheres are shown for each patient group. One can see that although the target for the deepest contact is similar in both PDD and DLB patients, the implantation trajectory in DLB patients was different, particularly for four of the electrodes where the top contacts were not optimised to be inside the GPi (see discussion).

### 3.3 LFP power

**Fig. 2:**
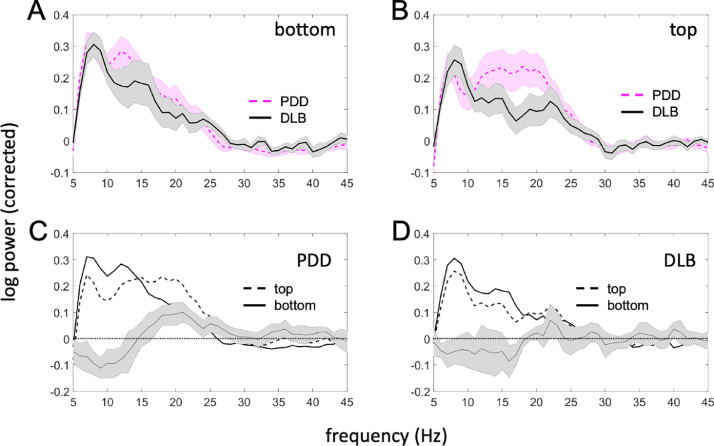
Comparison of oscillatory components of the power spectra between the top and bottom LFP channels and the two disease groups. (A) Spectra for the bottom (NBM) channel. (B) Spectra for the top (GPi region) channel. The shaded area shows standard error of the mean. (C) Comparison of top and bottom channels in the PDD group. (D) Comparison of top and bottom channels in the DLB group. The line with error bars shows the mean difference and its 95% confidence interval (paired comparison). The PDD patients appear to have increased beta power in the top, but not the bottom channel. There is also increased low frequency power in the bottom channel seen in both groups but more consistently in the PDD group.

Fig. 2 compares the spectra of the oscillatory component of LFP log-power between PDD and DLB patients, and between top and bottom contact pairs (corresponding to the GPi region and NBM respectively). Two effects were identified: (1) low frequency power (theta and alpha bands) was higher in the bottom (NBM) compared to the top (GPi region) channel, although this was not specific to disease. (2) beta power was higher in GPi region in PDD patients compared to DLB patients. Both effects were only significant at the uncorrected level (*p*<0.05) and would not survive multiple comparison correction across all frequencies. However, we chose to report them as both are consistent with prior literature (see Discussion).

### 3.4 Sensor-level coherence analysis

**Fig. 3:**
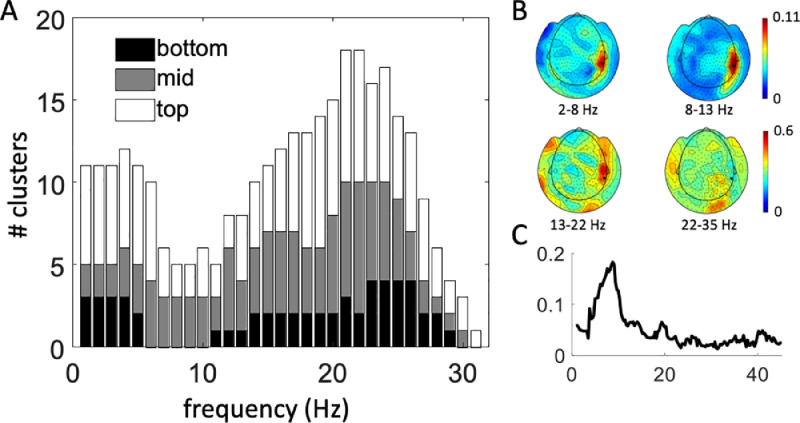
Sensor-level analysis of coherence. (A) Results of the sensor-level test for significant LFP-MEG coherence. Data is averaged across all patients and hemispheres within each frequency bin. For each frequency bin the number of significant clusters is shown separately for the bottom (01, NBM), mid (12) and top (23, GPi region) channels. (B) Example of coherence topographies in the bands used for source analysis for a single PDD patient (patient C). The coherence values were averaged over the three right LFP channels. (C) Coherence spectrum from a single MEG channel (MRT24, highlighted in panel B). A clear alpha-theta peak and a secondary smaller beta peak can be seen.

Fig. 3(A) shows the histogram of frequencies included in significant coherence clusters separately for the top, middle and bottom contact pairs. There are two separate clear peaks: in the delta/theta band (2–8Hz) and in the beta band (13–30Hz). Coherence is also present for some cases in the alpha band (8–13Hz) but there is no peak there. Overall, the frequency profiles look very similar for the 3 channels (there are just fewer contacts with significant coherence for the mid and bottom channels). Fig. 3(B) and (C) shows an example of sensor-level topographies of coherence and a single channel coherence spectrum respectively for one PDD patient (patient C).

### 3.5 Source-level coherence analysis

**Fig. 4:**
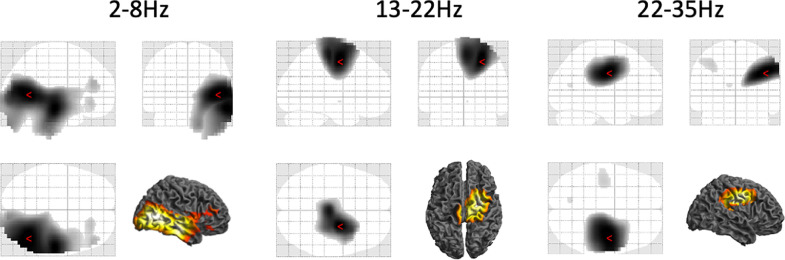
Results of between-frequency ANOVA on normalised DICS coherence images. Each band was compared to the average of the other 3 bands. The statistical maps were thresholded at *p*<0.05 FWE corrected at the peak level. The red markers represent locations with peak t-statistic values. No significant coherence specific to the alpha (8–13Hz) band was found.

Fig. 4 shows the results of between-frequency ANOVA on normalised DICS coherence images. Each band was compared to the average of the other 3 bands. In the theta band, coherence was with the entire ipsilateral temporal lobe with peaks at MNI coordinates 42, −62, 4 and 58, −22 −18. There was no significant coherence specific to the alpha band. In the low beta band the coherence was specific to the mesial sensorimotor areas peaking at MNI coordinates 24, −8, 52, and in the high beta band to the lateral sensorimotor areas (corresponding to the cortical hand representation of the sensorimotor cortex) peaking at MNI coordinates 42, −12, 34.

**Fig. 5:**
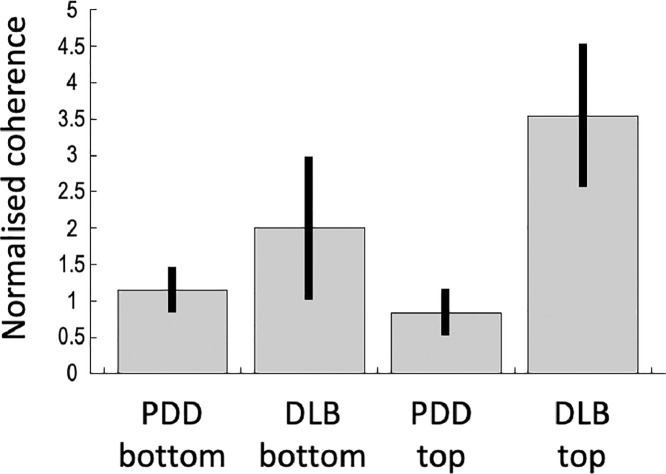
The results of follow-up ANOVAs testing for the effects of contact and disease for each band restricted to the areas of band-specific significant coherence. Contrast estimates and their standard errors are shown for the only significant effect which was an interaction between contact and disease in the high beta band (*p* = 0.036 FWE corrected) peaking at MNI coordinates 52, −2, 48 corresponding to Brodmann area 6. The interaction is driven by the coherence being higher in the DLB group in the top channel.

**Fig. 6:**
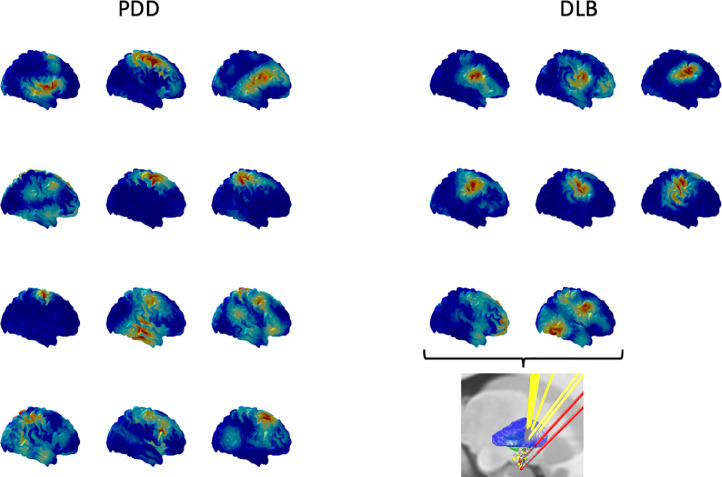
To further interrogate the interaction effect shown in Fig. 5, we plotted all the individual high beta coherence topographies for the top channel separately for the DLB and PDD groups. The colour scale is different for each map to be able to clearly see the differences in the topography. In the DLB group a consistent peak around the sensorimotor hand representation can be seen. The two hemispheres for which this peak is less clear (bottom two maps in the DLB group) come from the same patient (patient H) and correspond to the most outlying electrode trajectories (red in the inset) with the top contacts furthest away from the GPi.

Follow-up ANOVAs testing for the effects of contact and disease for each band restricted to the areas of band-specific significant coherence only found one significant effect which was an interaction between contact and disease in the high beta band (*p* = 0.036 FWE corrected) peaking at MNI coordinates 52, −2, 48 corresponding to Brodmann area 6. Fig. 5 shows the contrast estimates from the peak of this effect and one can see that the effect is driven by the difference between PDD and DLB which is specific to the top channel (GPi region). To further investigate the origin of this effect, we plotted the raw coherence topographies for all the individual hemispheres for the top channel separately for DLB and PDD (Fig. 6). For all the DLB hemispheres there was a coherence peak in the lateral sensorimotor cortex. This was not the case for PDD, where the topographies were more variable but tended to be more mesial and similar to the low beta band topographies for both diseases (not shown). In two of the DLB hemispheres the lateralised coherent pattern was not as clearly defined as in the other six. The corresponding electrodes came from the same patient (patient H) and examination of the lead locations in this patient (Fig. 6, insert) showed that their trajectories also differed in a way that placed the top contacts the furthest away from the others. Examination of the locations of these contacts with respect to the individual anatomy by an expert neurosurgeon placed them in the junction between the anterior putamen and the GPe (i.e. in the external medullary lamina).

### 3.6 Distinguishing between disease and spatial location effects

**Fig. 7:**
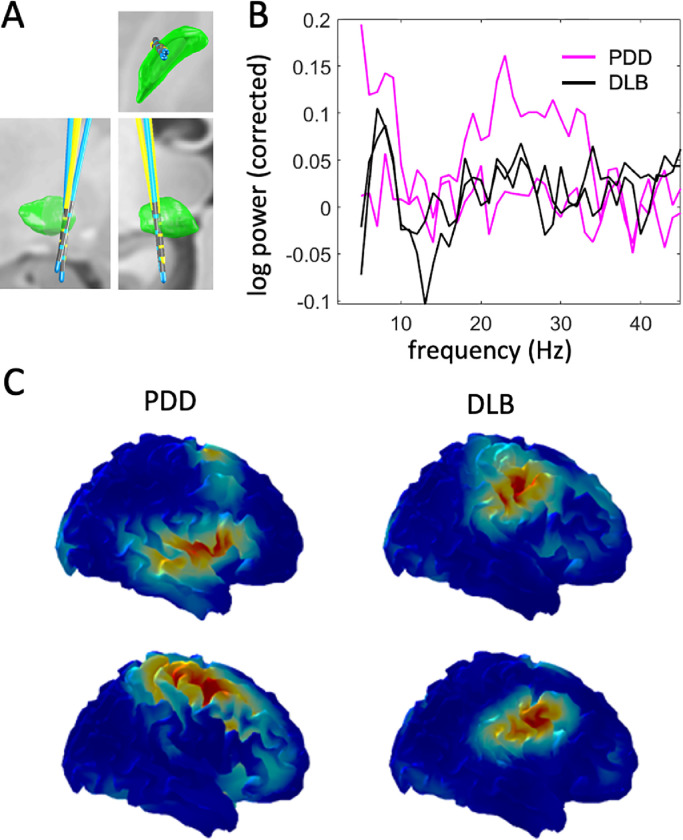
Comparison of two patients, one DLB (patient J) and one PDD (patient A) who had the most similar electrode trajectories. A. The electrodes for the PDD patient are cyan and the electrodes of the DLB patient are yellow. GPi boundaries are rendered in green based on the DISTAL atlas (Ewert et al., 2018). The view is axial from below (top), sagittal from the right (lateral) direction (bottom left) and coronal from the front (bottom right). B. Individual oscillatory components of the LFP spectra for the top channels in the four hemispheres corresponding to the electrodes in (A). Increased beta power can be seen in one of the hemispheres of the PDD patient consistent with the group effects. C. Comparison of normalized high beta coherence maps for the top channels. Each row corresponds to two channels for which the corresponding midpoints between the top two electrode contacts were the closest in the template space. Peaks around the sensorimotor hand area are seen in the DLB patient and not in the PDD patient.

Due to the small number of patients and the fact that the locations of the top contacts for DLB and PDD largely fall into two separate spatial clusters, it is not possible to do a systematic analysis across all patients to separate the factor of spatial location from that of disease. However, there was one DLB patient (patient J), for whom the electrode trajectories came very close to those of the majority of PDD patients. We, therefore, selected another pair of electrodes from the PDD group where the midpoint of the top contacts was the closest to the respective point for each of the two electrodes in this patient. The selected pair of electrodes turned out to come from the same patient as well (patient A). The distances within the two corresponding pairs of electrodes in these two patients were 1 and 1.2 mm (Fig. 7(A)). Comparison of the individual LFP spectra for the top channels in the four hemispheres showed that one of the hemispheres of the PDD patient showed clear increased beta compared to none in the DLB patient (Fig. 7(B). Comparing the high beta coherence topographies showed that both hemispheres of the DLB patient had a clear peak over the sensorimotor hand area while no such peaks were present in the PDD patient, where in one case the peak was more medial and in the other more lateral.

## 4 Discussion

In this study we combined electrophysiological and neuroimaging techniques to directly characterize resting state activity and cortical connectivity in the NBM and the GPi region in both PDD and DLB patents, and thereby investigate the relationship between these diseases. Our main findings were that both the NBM and the GPi exhibit patterns of long-range cortical synchrony. We identified three distinct coherent networks between the cortex and both the NBM and GPi: (1) A theta (2–8 Hz) band network between the NBM/GPi and temporal lobe, (2) a low beta (13–22 Hz) band network between the NBM/GPi and mesial sensorimotor areas, and (3) a high beta (22–35 Hz) band network between the NBM/GPi and lateral sensorimotor areas. Additionally, we observed differences in motor network activity in PDD and DLB in that: (1) there was significantly higher beta oscillatory power in the GPi region in PDD patients compared to DLB patients, and (2) we found a coherence pattern in the high beta band between the GPi region and lateral sensorimotor cortex that was more consistent across patients and hemispheres in the DLB group when compared to the PDD group. In the remainder of the discussion, we will first focus on the origins of the differences in motor network activity before discussing the observed resting synchrony profiles of the NBM and GPi.

There are a number of factors which could explain the observed differences in motor network activity in the two conditions. Firstly, the target for the deepest contacts in the DLB patient trial was a few millimetres more anteromedial in the Ch4i subsector of NBM compared to that used in our trial in PDD patients. As a result of this, the implantation trajectories were systematically different in most DLB patients compared to PDD, meaning that the most dorsal contacts, although still sited in the GPi region, were sited in different locations within and around that nucleus compared to in the PDD patients. The GPi is a large heterogeneous nucleus with complex sub-architecture (Parent and Hazrati, 1995) and consequently varying contact location by only several millimetres here could result in LFPs being recorded from entirely different neural subpopulations, with different functions and different cortical connectivity. Consequently, we cannot be sure that the GPi LFPs that we recorded from the PDD and DLB patients originate from the same neural subcircuitry in both conditions. This is, therefore, a potential confounding factor behind the differences in resting state motor activity that we observed, as these could be due to differences in contact location between the two diseases rather than due to true biologic differences. Although we could not address this issue in a statistical way due to the small patient number and systematically differing electrode trajectories in the two groups, we did compare two individual patients for whom the trajectories were the most similar (Fig. 7). This comparison – although with an N of only 4 hemispheres in 2 patients – tends to support the interpretation that increased beta power in the GPi region may be a more prominent feature of PDD, whilst high beta coherence with the hand area is a more prominent feature of DLB. Consistent with this is the observation that for the only DLB patient not showing as clear and focal high beta coherence patterns as the others, the electrode trajectories were different with the top contacts furthest away from the GPi (see inset of Fig. 6).

Secondly, the observed differences in motor network activity may be confounded by differences in the severity of motor symptoms in the PDD and DLB cohorts. Electrophysiological studies in Parkinson's disease (PD) patients have demonstrated that resting state beta oscillatory power in the subcortical motor nuclei (GPi and subthalamic nucleus (STN)) is the pathophysiological substrate of motor parkinsonism (Brown, 2003; Brown et al., 2001; Oswal et al., 2016a). Parkinsonian motor symptoms are more pronounced in PDD patients than in DLB patients of matched dementia severity (Petrova et al., 2015), indicative of less motor network dysfunction in the latter. The difference in UPDRS part III motor off scores between the PDD and DLB patients in our study sample is in line with this (Table 1). Therefore, given the lower level of motor network dysfunction in DLB compared to PDD, one would expect to find less resting beta oscillatory power in GPi in DLB patients compared to PDD patients, and our results support this. It should also be noted that our recordings were conducted on dopaminergic medication, which suppresses pathological beta oscillatory activity in subcortical motor nuclei in PD (Brown and Williams, 2005), such that the observed differences in beta power between the two groups were likely reduced compared to the off-drug state. It would have been preferable to perform the recordings off dopaminergic medications, but our patients were frail and probably would not have been able to tolerate this.

Regardless of the above confounds, the finding of a coherent network in the high beta band between the GPi region and lateral sensorimotor cortex more consistently seen in DLB patients is novel. Recent LFP-MEG studies in PD patients have demonstrated a similar coherent network specific to the high beta band between the STN and mesial sensorimotor cortex (Oswal et al., 2016a). This so-called ‘hyperdirect’ pathway in PD patients is the main driver of beta oscillatory power in the STN, and therefore crucial in the pathophysiology of parkinsonian motor symptoms. As one might expect, our PDD patients showed coherence in the high beta band within this same network. The fact that our DLB patients instead showed high beta coherence in a spatially distinct, more lateralised network is of uncertain significance. Our data unfortunately cannot reliably determine whether this lateral high beta band coherent network is specific to DLB, or related to either differences in electrode placement or motor function between the two patient groups. Further studies of resting state networks, ideally of a scalable non-invasive nature, are warranted to clarify this.

Our resting state LFP recordings from the NBM showed an oscillatory peak in the theta band. There were no significant differences in NBM oscillatory activity between PDD and DLB. Although low frequency power was slightly higher in the NBM than in the neighbouring GPi region in both diseases (Fig. 2, graphs C and D), this effect was not statistically significant after correction for multiple comparisons. Consistent with this finding, a previous study has reported low frequency power in the NBM to be greater than that in the GPi, in two PDD patients with implantation trajectories similar to those of our cohort (Nazmuddin et al., 2018).

NBM degeneration is key in the pathogenesis of both PDD and DLB (Grothe et al., 2014; Perry et al., 1985; Ray et al., 2018), however our clinical trials of low frequency NBM stimulation in these patient groups did not produce clear clinical effects (Gratwicke et al., 2020, 2018). Therefore, whether low frequency power in the resting NBM is physiological or pathophysiological, and its clinical relevance, remain to be determined.

Our coherence analysis did not show significantly higher coherence in the NBM compared to the GPi region in any frequency band in either PDD or DLB patients. The coherent network topographies we found for NBM in both conditions were also broadly similar to those reported in a previous LFP-MEG study of cortico-GPi coherence in patients with dystonia (Neumann et al., 2015). In terms of sensor-level profile of coherence frequencies (our Fig. 3, their Fig. 2(D)) the two studies give very similar results, with peaks in the beta band and low frequencies. Taken together, this could suggest that the coherent networks we observed are characteristic of the GPi rather than the NBM, and that, therefore, there is no NBM-specific coherent network, in line with previous work (Nazmuddin et al., 2018). This is not necessarily surprising as the NBM has anatomically diffuse efferent connections to most cortical areas (Gratwicke et al., 2013) while the GPi displays much more specific connectivity to particular cortical regions (Milardi et al., 2015), meaning that a discrete coherent cortical network is less likely in the former than the latter. However, the existence of two spectrally distinct cortico-NBM networks to temporal and sensorimotor cortex is also feasible and would be in keeping with recent anatomical studies that suggest functional segregation of NBM efferents to these structures (Gielow and Zaborszky, 2017; Záborszky et al., 2018).

Another limitation of this study is that we allowed the patients to take their usual doses of levodopa and AChEI medications on the day of the recordings. Administration of levodopa ameliorates pathological beta oscillations in subcortical motor nuclei in PD patients (Brown and Williams, 2005), which could, therefore, have partially masked the magnitude of differences in resting state activity seen in the GPi region in the PDD and DLB patients, as discussed above. Administration of AChEI medication could potentially have a similar masking effect on pathological neural activity in the NBM. However, given the degree of both motor and cognitive clinical disability that these patients experienced without these symptomatic medications, it would not have been possible for the patients to tolerate the recording sessions if the medications were withdrawn.

In conclusion, our study demonstrated similar patterns of local and long-range synchrony of both the NBM and the GPi in PDD and DLB. We also observed differences in resting state activity and cortical coherence in the GPi region between PDD and DLB patients. However, due to the small number of patients in this study and differences in electrode implantation trajectories between the patient groups it is difficult to say with certainty whether these observed differences truly represent different electrophysiological disease signatures or differences in the locations of recording contacts.

## CRediT authorship contribution statement

**James Gratwicke:** Conceptualization, Methodology, Investigation, Formal analysis, Writing - original draft. **Ashwini Oswal:** Investigation, Writing - original draft. **Harith Akram:** Resources, Writing - review & editing. **Marjan Jahanshahi:** Conceptualization, Supervision, Investigation, Writing - review & editing. **Marwan Hariz:** Conceptualization, Resources, Writing - review & editing. **Ludvic Zrinzo:** Conceptualization, Resources, Writing - review & editing. **Tom Foltynie:** Conceptualization, Methodology, Investigation, Supervision, Resources, Project administration, Funding acquisition, Writing - review & editing. **Vladimir Litvak:** Conceptualization, Methodology, Investigation, Software, Resources, Formal analysis, Data curation, Visualization, Writing - original draft.
